# Opinions on Dental Erosive Lesions, Knowledge of Diagnosis, and Treatment Strategies among Norwegian Dentists: A Questionnaire Survey

**DOI:** 10.1155/2012/716396

**Published:** 2012-08-15

**Authors:** Aida Mulic, Simen Vidnes-Kopperud, Anne B. Skaare, Anne Bjørg Tveit, Alix Young

**Affiliations:** ^1^Department of Cariology and Gerodontology, Institute of Clinical Dentistry, University of Oslo, P.O. Box 1109, Blindern, 0317 Oslo, Norway; ^2^Department of Pediatric Dentistry and Behavioural Science, Institute of Clinical Dentistry, University of Oslo, P.O. Box 1109, Blindern, 0317 Oslo, Norway

## Abstract

This study aimed to investigate dentists' general experience, knowledge about diagnosis, and treatment of dental erosive wear in young adults. A questionnaire was sent to 1262 Norwegian public dental health-employed dentists. The response rate was 60%. Results indicated that most dentists recorded erosive wear, half of them used a specific scoring system, and half registered lesions at the tooth surface level. Lesions were reported most often on palatal surfaces of upper anterior teeth (79% of dentists), on occlusal surfaces of lower 1st molars (74%), and on upper 1st molars (32%). Half the dentists used clinical photographs for documentation and 60% made study models. While 40% reported more erosive lesions in males, 36% reported no gender differences. High intake of carbonated beverages and acidic juices were reported as the most common cause by 97% and 72% of the dentists, respectively. Only 21% of dentists recorded the patient's dietary history, and 73% never measured saliva secretion. The majority (78%) of the dentists treated patients with erosive wear themselves. In general, the survey suggests that the dentists are relatively up to date regarding the clinical recording, diagnosis, and treatment of dental erosive wear. However, dietary and salivary analyses were not given priority, and early, preventive treatment was lacking.

## 1. Introduction

The focus on dental erosive wear by both the public and dental health professionals appears to be increasing. This may be due to a decrease in caries levels in industrialized countries [1] and/or to high prevalence of erosion lesions documented in recent epidemiological studies [2–4]. The diagnosis and the potential treatment needs of patients with dental erosive lesions are considered to be a significant challenge to clinicians [5].

It is known that dental erosive wear is a multifactorial condition [6], and that it is important to evaluate the potential factors which could lead to identification of persons at risk. In order to assess and reveal possible predisposing factors, a record of patient's dietary intake should be registered as part of a comprehensive case history involving general health, habits, saliva flow rates and buffer capacity [5]. A study by Lussi and Schaffner showed that active lesions will progress when no adequate preventive measures are implemented [7]. It is therefore important to detect the condition as early as possible and dentists should be trained to register even the first signs of the condition. This becomes especially important when taking into account the trend that patients often do not seek treatment until the lesions have reached such an advanced stage that restorative therapy is needed [8]. Furthermore, the clinical appearance of the eroded tooth surfaces is the most essential sign since there are no diagnostic tools available for early, quantitative detection. The clinical examination of the teeth should be performed systematically using a simple and accurate scoring system [8].

The scientific literature shows that there has been an increasing focus on the aetiology, prevalence, and treatment options for dental erosive lesions. However, a survey among general dental practitioners from 2003 showed that a large proportion of dentists advised their patients about the erosive wear only occasionally or rarely [9]. Of the 1686 12 year olds included in that study, less than 10% could recall their dentist mentioning the condition. Recent studies have also identified that an inadequate level of information about dental erosive wear was given by dental practitioners to adolescents/adults in possible risk groups [10, 11]. Furthermore, another recent study showed that the knowledge and awareness about dental erosion among dental students, faculty members and patients in a Brazilian dental school were poor [12].

Therefore, the aim of the present study was to perform a survey on public dental practitioners asking them about their experiences, awareness, knowledge of diagnosis, and choice of treatment options for dental erosive wear in 18- to 30-year-old patients.

## 2. Material and Methods

**Figure 1:**
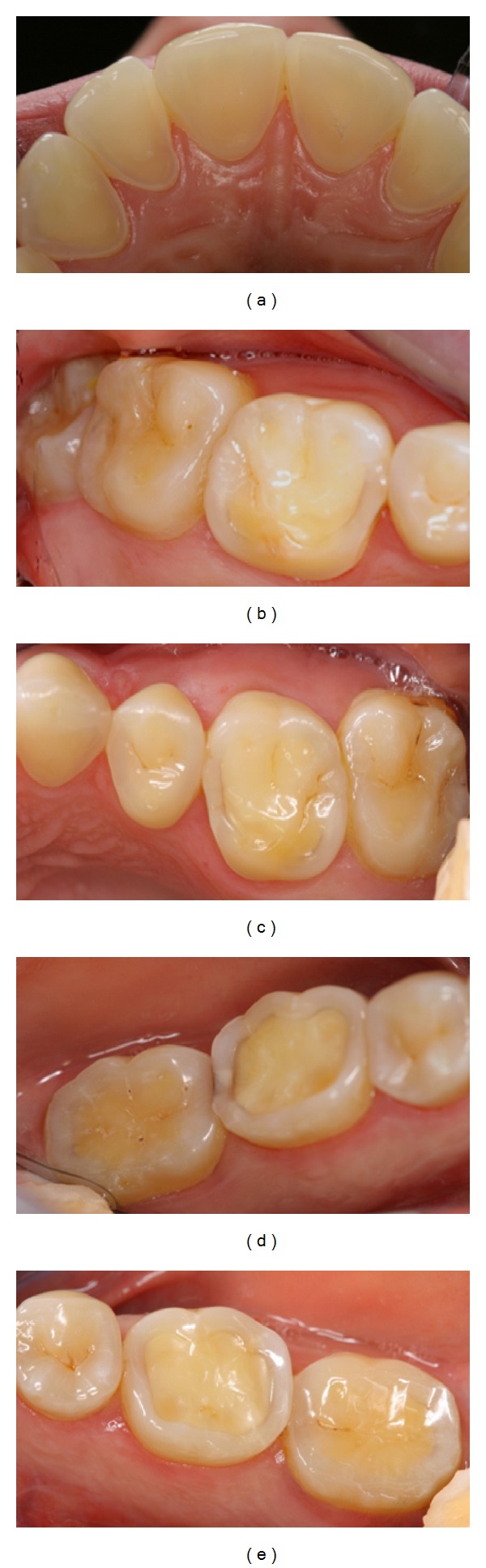
Clinical photographs of the patient case: A 28-year-old woman who had an eating disorder with vomiting as a teenager, but is now healthy. (a) palatal surfaces of the upper incisors, (b) occlusal surfaces of upper right 1st and 2nd molars, (c) occlusal surfaces of upper left 1st and 2nd molars, (d) occlusal surfaces of lower right 1st and 2nd molars, and (e) occlusal surfaces of lower left 1st and 2nd molars.

A precoded questionnaire was sent electronically to all dentists (*n* = 1262) employed in the Public Dental Health Service (PDHS) in Norway in April 2011, using the Internet-based software program QuestBack. Information was collected on the respondents' sex, age, home county, and type of dental practice, and to which extent the respondents were involved in the diagnosis and treatment of dental erosive wear. The questionnaire had two parts. In one part the dentists were asked how they register and document dental erosive wear in their patients aged 18–30 years. They were also asked questions relating to their experience of erosive lesions, such as their estimation of the distribution and prevalence of erosive lesions among these patients. The dentists' opinions on probable causes related to erosive lesions were also recorded. The second part of the questionnaire involved a patient case, where the dentists were asked to record their choice of treatment including general patient advice and/or type of restoration. A brief patient history as well as colour clinical photographs of labial and palatal surfaces of upper anterior and molar teeth with differing severity of erosive lesions was provided (Figure 1). The questions are described below.

**Patient Case**

A 28-year-old woman who had an eating disorder with vomiting as a teenager, but is now healthy.

**Question One**

What type of advice would you give this patient (you can make more than one choice)? (1) Information about dietary and drinking habits; (2) information about brushing technique/habits; (3) recommend rinsing with fluoride; (4) recommend rinsing with chlorhexidine; (5) recommend fluoride tablets; (6) refer to specialist, faculty clinic, or other dentist; (7) recommend specific toothpaste or rinse.

**Question Two**

How would you treat the teeth in the maxillary anterior and posterior regions and the mandibular posterior teeth (you can make more than one choice)? (1) No treatment; (2) treat locally with fluoride solution (e.g., 2% NaF, Duraphat, Fluor Protector); (3) apply bonding material; (4) apply flow composite; (5) restore with glass ionomer cement; (6) restore with composite filling material; (7) restore with compomer; (8) restore with ceramic laminate/facet/inlay/onlay; (9) restore with crown.

The QuestBack program was configured to automatically send reminders to all participants who had not replied after 4, 7, and 12 weeks.

### 2.1. Statistical Analysis

The data was processed using SPSS version 18.0.3 (Statistical Package for the Social Sciences; SPSS Inc., Chicago, Ill., USA) and statistical evaluation was carried out by means of descriptive statistics. As the upper 1st molars were most commonly suggested for operative treatment by the dentists, multivariate logistic regression analyses were performed to test the association between operative treatment of these teeth (dependent variable) and the following independent variables: dentists' age, gender, practice location, and choice to refer. Unadjusted results were obtained by performing separate regression analyses for each selected variable. Adjusted results were obtained by including all the selected variables in one regression analysis. In the adjusted analysis the result for each variable was adjusted for all the other variables. Results were reported using odds ratio (OR) with 95% confidence interval (CI) and *P* value (P). The probability level for statistical significance was set at *α* = 0.05.

### 2.2. Ethical Considerations

Participation was voluntary and no compensation was given to the respondents. Anonymity was ensured by QuestBack. The study was approved by the Norwegian Social Science Data Services.

## 3. Results

### 3.1. Details about the Respondents

A total of 1262 dentists were invited to participate in the study, and 783 dentists had responded after three reminders. Respondents who stated that they did not normally work with patients having dental erosive wear (*n* = 78) were excluded from the statistical analyses. A response rate of 60% was calculated according to Standard Definitions of the American Association for Public Opinion Research [13]. The respondents ranged in age from 25 to 72 years (mean 43.3 years, SD 13 years) and consisted of 65% females and 35% males.

### 3.2. Descriptive Analyses

Almost all respondents (*n* = 695, 98.6%) registered dental erosive lesions in their patient's charts. Of these, 50% used a specific scoring system: 31% used a two-graded scoring system (enamel dentine) and 14% used a more detailed system, while 5% did not report details on their scoring system. Erosive lesions were registered at the surface level by 58% of the respondents, at the tooth level by 15%, and at the individual level by 26%, while 1% did not answer the question. Documentation with clinical photographs was reported as being often performed by 9% of the dentists, sometimes by 51%, and never by 40%. The corresponding values for documentation with study models were 5% (often), 60% (sometimes), and 35% (never). The dentists reported that the erosive lesions were most often located on the palatal surfaces in upper anterior teeth (79%), followed by first mandibular molars (74%) and first maxillary molars (32%). According to 36% of dentists there were no gender differences in the prevalence of erosive lesions. While 40% of the dentists reported that erosive lesions were more common in males than in females, only 4% of the dentists stated that more females than males had erosive lesions, and 20% were unsure.

Of the dentists with more than 10–15 years clinical experience (*n* = 404), the majority (81%) reported a higher prevalence of erosive lesions today compared with 10–15 years ago. Eight percent of the dentists did not have the impression of seeing more erosive lesions today, while 11% were unsure.

Half of the dentists (52%) did not think that young adults (aged 18–30 years) with dental erosive lesions had more caries than those without erosive lesions. Higher caries prevalence amongst patients with erosive lesions was estimated by 19% of dentists; 19% stated no differences and 10% were unsure.

Most of the dentists (77%) reported that they usually found a probable cause of the erosive lesions, 22% occasionally, and only 1% reported that they seldom found a probable cause. The most common causes given were consumption of carbonated beverages (97%), followed by acidic juices (72%), fruits (46%), sport drinks (24%), and acidic diet (20%). Reflux and eating disorders with vomiting were reported as more uncommon causes (8%) by the dentists.

When patients were registered as having erosive lesions, 21% of the dentists always recorded a dietary history. A quarter of the dentists (24%) reported that they often recorded a dietary history, 36% only occasionally, and 19% reported that they never recorded dietary history in patients registered with erosive lesions. The type of dietary questionnaire used was highly variable; 51 dentists (9%) used a precoded questionnaire, 31% asked the patient to record all they consumed during a certain period of time (amount, time of day, etc.), while the remaining 60% used different techniques, such as oral interviews and describing text in the dental chart.

More than half of the respondents (57%) estimated that their patients with erosive lesions had a normal amount of saliva, while 37% did not know. Only a small percentage of dentists estimated that their erosion patients had less than normal saliva flow (4%) and 2% thought they had more than normal amounts of saliva. Only two dentists (1%) reported that they always measured saliva secretion, 1% reported measuring saliva flow often, 25% only occasionally, while 73% reported that they never measured saliva secretion in patients with dental erosive lesions.

Most of the dentists (78%) treated their patients with dental erosive wear themselves, while 9% referred the patients to another dentist or specialist/university faculty clinic. The remaining 13% treated many patients themselves, but referred the more severe cases. The two latter categories were combined into one variable when performing multivariate analyses. Eight percent of all the male dentists would refer all patients with erosive lesions to another dentist or specialist/university faculty clinic, compared with 10% of the female dentists. The mean age was 44.6 years (males) compared with 43.3 years (females) among the dentists who treated their patients themselves. Significantly more dentists living in the eastern part of Norway (12%) referred patients compared with dentists living in the northern (7%) and western (7%) parts of Norway (*P* = 0.03).

### 3.3. Patient Case

A previously calibrated examiner scored the severity of the erosive lesions on the surface level based on the clinical photos [14] using a tested scoring system for erosive lesions (VEDE system) [14]. Enamel lesions (grade 2) were registered on central incisors and upper 2nd molars, while dentine lesions (grades 3, 4, and 5) were seen on lateral incisors, all 1st molars and lower 2nd molars, indicating a case with severe erosive lesions (Figure 1).

The results are presented in Tables 1 and 2. The majority of dentists (88.2%) would give the patient information about good dietary and drinking habits and recommend that the patient should rinse with fluoride (87.1% of the dentists). More than half the dentists (58.4%) would give information about tooth-brushing technique and/or brushing habits. Operative treatment was the most common choice of treatment for the upper 1st molars. Forty-four percent of the dentists chose to place a filling, and 18.8% chose prosthodontic treatment for these teeth. The lower 2nd molars were the most likely teeth to receive either no treatment other than advice (35.3%) or local treatment with fluoride solution/use of a bonding material (40.6%). These teeth were also least likely to be restored with a filling (20.1%). There were no obvious differences between the treatments chosen for the different upper incisor teeth, and most dentists would choose to restore them with a filling.

The results of the multivariate logistic regression analyses are shown in Table 3. Only the gender of the dentist remained significant in the analysis. An odds ratio of 1.5 indicated that for upper 1st molars, the female dentists suggested operative treatment significantly more often than the male dentists.

## 4. Discussion

Even though there are few comparable studies, this survey tends to suggest that the majority of dentists are aware of the problem of dental erosion and are relatively up to date regarding the clinical recording and diagnosis of dental erosive wear. The major points to note are that the documentation of erosive dental wear was not standardized, and little or no priority was given to dietary and salivary analyses. Despite this, and more encouragingly, the majority of dentists reported that they were confident of finding the cause of the erosive wear and also in treating their own patients that need reparative therapy due to dental erosive wear.

The questionnaire used in this study was aimed at dentists working in the Norwegian PDHS who were currently involved in the diagnosis and treatment of dental erosion in their clinical practice. In general, these dentists see a large proportion of adolescents and young adults, and based on recent prevalence studies this patient group may be considered as being at high risk for dental erosion [3, 4, 15, 16]. Importantly, responding dentists who reported that they did not normally work clinically with erosive dental wear were excluded from the study.

Measures were taken to ensure a high response rate in accordance with a systematic review of questionnaires [17]. The questionnaire was styled in a personal manner, and a realistic and illustrative case with background information was presented early in the questionnaire, as was proven successful in a previous questionnaire-study on dental caries [18]. In order to emphasize the importance of a reply, when sending reminders to the dentists, respondents were informed of the response rate so far for the questionnaire. Unfortunately, as this questionnaire was anonymous, no information could be retrieved about the nonresponders, and a meaningful nonresponse analysis could not be performed. Another limitation of this study is that such questionnaires are based on the respondents' ability to recall. Furthermore, given that the specificity and sensitivity of the dentists' diagnosis of erosive wear were not assessed, the accuracy of the data cannot be quality controlled.

Recent prevalence studies have shown that erosive tooth wear is a common problem in current dental practice and is a challenge for the practitioner [3, 4, 12, 15, 16]. In the present survey, the dentists were primarily asked to report on dental erosive wear on younger individuals. The majority of dentists participating in this study reported a higher prevalence of erosion lesions today, compared with 10–15 years ago. Even though lesion due to the combination of erosion and abrasion/attrition is a common phenomenon, one can expect that the presence of mainly erosive wear lesions exceeded the number of erosion/abrasion/attrition lesions in the age group included in this study [19].

To be able to record the presence and severity, as well as the progression of erosive lesions, use of a grading system is required [20]. In the present study, the majority of the dentists reported that they used a specific scoring system registered at the tooth surface level. In most cases this was the registration system included in the electronic patient journal system. Approximately one quarter of the dentists registered the erosions at the patient level only, which does not allow for specific followup of lesion progression. Regarding the oral distribution of the erosive lesions, the most commonly affected tooth surfaces were the palatal surfaces of the upper incisors and the occlusal surfaces of the lower 1st molars. The occlusal surfaces of upper 1st molars were also relatively frequently affected. These results are in accordance with many previous studies in adolescents [10, 21, 22].

Even though approximately one-third of dentists had the impression that there were no differences between the sexes, 40% reported more dental erosive wear in males. Several studies have reported a higher prevalence of dental erosion in male adolescents [2, 4, 15, 22]. In a recent study the consumption of soft drinks and other acidic beverages was found to be related to both age and gender [23]. Boys consumed significantly greater amounts than girls. One could expect that a higher intake of such beverages (often containing sugars), typically consumed between meals, could also lead to more dental caries. While half the respondents in the present study thought that patients with dental erosion did not have more dental caries, about one fifth of the dentists reported a higher incidence of caries in these patients, and about the same percentage reported that patients with dental erosion had the same amount of caries as patients without dental erosion. Results from other studies regarding presence of dental erosive wear and caries experience are also ambiguous. Some studies have demonstrated a significantly higher caries experience among children with high prevalence of dental erosion [24, 25], whereas others could not verify this association [26, 27].

In order to identify possible aetiological factors in patients presenting with dental erosion, a dietary history is extremely important, as behavioural factors such as eating and drinking habits play a key role in the pathogenesis [28]. Only half of the dentists in this study reported either occasionally or never, recording a dietary history in patients with dental erosion, and only a small proportion used a precoded questionnaire. This is a worrying finding, as the effective prevention of progression of erosive lesions often requires clear, individualized dietary advice.

Apart from behavioural factors, biological patient factors also play an important role in the pathogenesis of dental erosion [28]. It is well known that a high salivary flow rate favours the prevention or minimization of initial acid attack [29]. In the present study the majority of dentists reported that they did not measure saliva secretion in their dental erosion patients. This reflected the estimate by more than half the dentists that their patients had normal amounts of saliva. However, more than one-third of the dentists reported that they did not know about the saliva status of their dental erosion patients, possibly indicating a lack of knowledge about the importance of saliva in the aetiology of dental erosive wear. However, if the large majority of patients seen by these dentists are adolescents or young adults, the percentage of patients presenting with subnormal levels of saliva secretion is not expected to be very high.

In the present study, approximately two-thirds of the dentists reported that they usually found a probable cause of the dental erosion in their patients. Nearly all dentists reported that high consumption of carbonated soft drinks was the most likely cause. Acidic drinks are thought to be one of the most important factors leading to dental erosion, especially when taking into account that the consumption of such drinks has increased greatly over the last decades [30, 31]. Norway is no exception, with a yearly mean consumption of well over 100 liter per person, placing them in the top five among the European countries. Frequent intake of fruit and/or fruit juices was also commonly reported by the dentists to be causative factors in their patients. The risk of developing erosive tooth wear was found to be 37-times higher in persons with a greater than twice daily intake of citrus fruits than in persons eating fruit less often [32].

Dental personnel may in many cases be the first health workers to observe signs of eating disorders. In the present questionnaire dentists regarded reflux and eating disorders with vomiting as relatively uncommon causes of dental erosion in their patients. While no difference was reported in the prevalence of dental erosion between young Icelandic adults and patients with gastroesophageal reflux disease (GERD) [31], a more recent study found that patients with eating disorders (mean age 21 years) were at 8.5-times higher risk of having dental erosion [33].

Thorough documentation of the patient case is a standard part of following the progression of erosive lesions and treating patients with erosive tooth wear. For patients where the degree of damage suggests the need for extensive rehabilitation, insurance companies or social security systems usually expect solid documentation. In the present study, the majority of dentists only occasionally documented their patient cases by taking clinical photographs or making study models. Surprisingly, more than a third of the dentists never used this type of documentation. This may be related to the possible mild severity of the patient cases or the fact that they are not aware of the importance of documenting such wear.

A 3-year longitudinal study on the dynamics of tooth erosion in adolescents concluded that the condition progressed steadily in children with dental erosion [34]. At the same time, there are reports that according to patients, dentists do not appear to give enough information about this problem. In a recent study examining the status of the dentition in professional Norwegian wine tasters, seven of the nine wine tasters registered with dental erosive wear had not been informed by their dentist/dental hygienist about the presence of these lesions [11]. In another study examining dental erosion in a group of individuals training regularly at a training studio, 82% of the physically active young adults with erosive wear who recently had been to their dentist/dental hygienist claimed they had not been informed about the presence of these lesions [10]. Furthermore, in the study on dental students, faculty members, and patients in a Brazilian dental school, Hermont and coworkers [12] have recently shown that knowledge and awareness about dental erosion are poor, and they concluded that there was a need for better understanding and communication. Maybe it is still true that many dentists typically ignore or overlook the very early stages of erosive tooth wear, dismissing tooth surface loss as a normal and inevitable occurrence of daily living, and thus not worth spending time on any specific interventive activity, as was suggested a few years ago [5].

In the present survey most of the dentists reported that they perform the reparative treatment of their own patients with dental erosion, and only nine percent referred the patients to another dentist, a specialist or a university faculty clinic. There were no significant age or gender differences with regard to this aspect, an observation that was made in a similar clinical decision-making questionnaire among prosthodontists [35]. This indicates that most of the general dentists in this study feel competent in their technical skills. However, with respect to treatment options, significantly more of the female dentists who responded to the present questionnaire chose to restore the upper 1st molars. Regarding referral patterns, not surprisingly a greater proportion of dentists in the eastern, more populated part of Norway, where there are many specialists, referred their patients for restorative treatment, compared with those located in the northern and western parts of Norway, where there are few specialists.

The patient case included in the current study is not an uncommon example of a contemporary young adult. Prior registration of the erosive damage by one of the authors indicated that the patient had a combination of mild enamel and severe dentine lesions. The results of the questionnaire suggest that the responding dentists had few problems registering this difference. The 1st upper molars were regarded as the teeth with the greatest need for a restoration, probably because in this patient the palatal cusp was almost completely worn away. However, also the lateral incisors, upper 2nd molars, and central incisors were recommended to be filled by over a third of the dentists. Surprisingly, even though the lateral incisors and lower 1st molars had exposed dentine, more than a quarter of the dentists suggested that no restorative treatment was necessary. In contrast, about two-thirds of the dentists would restore enamel lesions on central incisors and 2nd molars using resin composites. This inconsistency could be a result of the difficulty for dentists in differentiating between more severe enamel lesions and early dentine lesions, as discussed in the literature [36]. Another consideration could be related to dentists' focus on the tooth level rather than considering the patient case as a whole.

In a similar questionnaire-based study [35], for a patient case involving palatal erosion in maxillary incisors without loss of the incisal edges, most specialists chose the prevention option that involved covering the worn surfaces with a dentine bonding agent and prescription of fluoride mouthwash. Restorative management in the form of direct composites was most commonly chosen by UK dentists while the non-UK prosthodontists were more divided, half of them choosing to leave the teeth untreated, about one-third choosing composites, and almost 10% choosing to crown the teeth. In a recent study performed on dentists in the Dental Practice-Based Research Network, direct resin-based composites were the most common type of restorations placed in noncarious tooth defects [37]. Although the patient group in that study was older than the average age of patients seen by the dentists in the present study, the choice of resin composites is a common factor.

Several authors have published strategies for the restorative treatment of patients with dental erosive tooth wear. Some clinicians have suggested that adequate function and aesthetics can be achieved when dental erosion lesions are restored using a combination of bonded ceramic overlays to reestablish vertical dimension and composite resin to restore the worn palatal and incisal surfaces of the upper anterior teeth [38]. Recently a new classification system (ACE classification: anterior clinical erosion), strictly related to the clinical observation of the status of eroded anterior maxillary teeth affected, was proposed [39]. Minimally invasive techniques with composite were recommended to restore the palatal surfaces, while the facial surfaces were recommended to be treated with ceramic veneers when necessary. The authors concluded that the question of whether it is preferable to start earlier with a lighter, less invasive rehabilitation or later with a highly aggressive but eventually more resistant one is still open for debate. In all these studies it would appear that direct composite restorations are considered to be the main approach in less severe cases.

## 5. Conclusion

Despite the limitations of gathering information using a survey of this type, the results tend to suggest that the responding dentists are relatively up to date regarding the clinical recording and diagnosis of dental erosive wear, although dietary and salivary analyses were not given priority. Furthermore, although documentation was not standardized, the majority of dentists reported that they felt confident of finding the cause of the erosive wear and of treating their patients themselves. As dental erosion appears to be increasing in prevalence, one would expect that newly trained dental health personnel will need to be even more aware of the necessity of careful and early diagnosis of this problem and that they will be even better equipped to face the future clinical challenges related to dental erosive wear.

## Figures and Tables

**Table 1:** The frequency (%) of dentists' general patient advice(s) in the patient case. *n*: number of dentists responding to each question.

Advice	%
Information about good dietary and drinking habits	88.2
Information about brushing technique/habits	58.4
Recommend rinsing with fluoride	87.1
Recommend rinsing with chlorhexidine	0.4
Recommend fluoride tablets	14.8
Refer to specialist, faculty clinic, or other dentist	9.0
Recommend specific toothpaste or rinse	11.2

**Table 2:** The frequency (%) of dentists' general choice of treatment and/or type of restorative material in the patient case. *n*: number of dentists responding to each question.

Treatment decision (%)	Incisors		1st molars		2nd molars	
	Centrals	Laterals	Lower	Upper	Lower	Upper
	*n* = 705	*n* = 656	*n* = 681	*n* = 680	*n* = 648	*n* = 649
No treatment	28.1	26.4	26.1	16.0	35.3	22.3
Treat locally with fluoride solution or bonding material	29.6	27.3	31.0	20.9	40.6	28.5
Restore with filling	34.9	38.7	34.0	44.3	20.1	36.1
Restore with ceramic laminate/facet/inlay/onlay	5.4	5.8	4.6	8.8	1.7	5.4
Restore with crown	2.0	1.8	4.3	10.0	2.3	7.7

**Table 3:** Associations between selected variables and operative restoration of upper 1st molars. Results are presented as odds ratios (OR) with 95% confidence interval (CI). Results significant at 5% level are marked in bold. ref: reference category.

Selected variables	Unadjusted			Adjusted		
	OR	95% CI	*P* value	OR	95% CI	*P* value
Age						
43–72 years (ref)						
25–42 years	1.1	0.8–1.5	0.71	1.2	0.9–1.7	0.31
Gender						
Male (ref)						
Female	**1.4**	**1.0**–**2.0**	**0.03**	**1.5**	**1.1**–**2.1**	**0.02**
Practice location						
East (ref)						
West	1.1	0.8–1.6	0.53	1.1	0.8–1.6	0.55
North	0.8	0.5–1.2	0.29	0.8	0.5–1.3	0.36
Refer cases						
No (ref)						
Yes	1.2	0.7–2.0	0.56	1.2	0.7–2.1	0.5
